# Binaural localization of musical pitch using interaural time differences in congenital amusia

**DOI:** 10.1371/journal.pone.0204397

**Published:** 2018-09-21

**Authors:** I-Hui Hsieh, Ssc-Chen Chen, Jia-Wei Liu

**Affiliations:** Institute of Cognitive Neuroscience, National Central University, Jhongli County, Taoyuan City, Taiwan; University College London, UNITED KINGDOM

## Abstract

Naturally occurring sounds are routinely periodic. The ability to phase-lock to such periodicity facilitates pitch perception and interaural time differences (ITDs) determination in binaural localization. We examined whether deficient pitch processing in individuals with congenital amusia (tone deafness) is accompanied by impaired ability to lateralize musical pitch at auditory periphery and memorize the location of pitch at the working memory level. If common mechanisms subserve processing of temporal-fine-structure based pitch and ITDs, then deficient processing of one feature should impair performance on the other. Thus, we measured ITD discrimination thresholds using an adaptive-tracking procedure for lateralizing musical tone pairs separated by different semitone intervals. Amusic individuals exhibited normal ITD thresholds comparable to those of matched controls, which were not affected by concurrent pitch changes. For working memory tasks, the amusic group performed significantly worse than matched controls in probed pitch recall, irrespective of the complexity level of concurrent variations along the ITD dimension of the melodic sequence. Interestingly, despite normal peripheral ITD thresholds, amusic individuals performed worse than controls in recalling probed locations of tones within a sequence of musical notes originating from different ITD-simulated locations. Findings suggest that individuals with congenital amusia are unimpaired in temporal fine-structure encoding to determine the location of musical pitch based on binaural ITD information at the auditory periphery. However, working memory for a sequence of sounds’ ITD-dependent spatial location is here shown to be impaired and dissociated from the pitch feature of sounds at the working memory level.

## Introduction

Most naturally occurring sounds, including musical pitch, occur at a restricted location in space, so encoding the sound object may implicate both pitch and location information. One important cue for the listener in extracting these sound features relies on the ability to detect the periodic structure of the acoustic waveform. Encoding the sound’s spectro-temporal oscillation pattern serves important functions in both binaural sound localization and pitch processing. The binaural cycle-to-cycle disparities present in the temporal fine-structures of acoustic stimuli provides interaural time difference (ITD) information, the primary cue used for localizing sounds in space [1–4]. Phase-locking pattern to the temporal-fine-structure of acoustical signals is also used by the auditory system in determining a sound object’s pitch at the peripheral level, at least for low-frequency tones (i.e., < 5 kHz). Previous research have suggested that efficiency in encoding the temporal-fine-structure of sound waveforms should determine both pitch perception and ITD lateralization performance based on common underlying neural temporal mechanism [5–8].

Here we considered the special case of a population affected by severe impairment in processing pitch. Commonly referred to as tone-deafness, congenital amusia is a neurodevelopmental auditory disorder characterized by deficits in pitch perception and production that cannot be attributed to hearing loss or neurophysiological causes [9]. This lifelong condition has been estimated to affect 1.5%–4% of the general population with slightly higher rates in females [9–11], has been shown to be hereditary [12, 13], and does not appear to co-occur with other cognitive disorders [11]. Besides having pitch discrimination difficulties [14–16], individuals with congenital amusia are impaired in pitch contour identification [17, 18], melodic sequence recognition [19], singing in tune [20], as well as in memorizing pitch-based materials [14, 21–23]. However, amusics show normal processing on some other music related attributes such as musical emotion based on temporal or timbral cues [24], but not when emotion was elicited by the tonality of musical excerpts [25]. Recent theories have suggested that amusic individuals’ pronounced difficulties in pitch processing may stem from an inability to use the fine spectro-temporal cues in the resolved harmonics in complex tones without peripheral basis [26]. Consistent with this view, one recent meta analysis has shown that pitch change is an effective moderator of the effect size of performance gap between amusic and control across studies, supporting the hypothesis that amusia stems from a broad disorder of acoustic pitch processing [27].

While much has been discovered about perceptual and memory impairment in amusics, the ability to lateralize musical pitch spatially has been less explored. Previous studies on the relationship between musical pitch and spatial processing in amusics have been investigated using primarily visuo-spatial materials, such as imagined transformations of hand-drawn figures, mapping pitch onto vertical spatial configuration, or three-dimensional mental rotation tasks presented visually [28–30]. These studies have reported inconsistent results regarding whether pitch impairments transfer to affect spatial processing ability in amusic individuals [29, 30]. The contribution of ITD, the most prominent binaural cue, in lateralizing sounds in space could be more susceptible to the influence of an impaired pitch processing system (than visual-spatial processing). As encoding ITDs has been hypothesized to rely on the same specialized mechanism as encoding periodicity and pitch [6–8], an inability to use fine spectro-temporal information could affect spatial hearing ability [8, 26]. However, only one recent study has directly examined spatial processing in amusics by measuring the difference limens in tracking which interval of consecutive bursts of low-pass noise contained the moving sound sensation induced by ITD or ILD cue [26]. One potential explanation that equivalent sensitivity to ITD information contained in low-pass filtered noise was reported between the amusic and control group in this study could be because low-pass noise can be considered as a “reduced pitch” or non-pitch situation. We expect that lateralization of musical pitch based on ITD cue could be more susceptible to the interference due to the coding “noise” associated with musical pitch processing in amusic individuals.

The idea that dysfunction at low-level sensory processing affects perceptual discrimination and short-term memorization of sounds in congenital amusics has been indicated in several recent studies. Specifically, several studies have reported that amusic individuals showed impaired performance on pitch retention tasks which decreased as a function of the physical pitch distance, suggesting that the level of difficulty associated with pitch discrimination affects pitch memory [31–34]. Also, one study reported that decreasing the amount of time given to encode tones impaired pitch discrimination and memory performance in congenital amusia compared to controls [31]. Similar effects have been observed in typical listeners and related neurodevelopmental auditory disorders, suggesting an interdependent relationship between discrimination and working memory performance, both of which are affected by abnormalities in early-steps of auditory processing [32, 34–36]. We hypothesized that an impaired spectro-temporal processing system in amusics could not only affect discrimination of ITDs in musical pitch at the perceptual level, but also when memorizing the ITDs of musical note sequences.

In this work, we examine whether an impaired use of fine spectro-temporal information in encoding sounds in congenital amusics affects lateralizing musical pitch at the discrimination and working memory level using ITD cues. To our knowledge, no prior studies have investigated memory of musical pitch’s locations based on binaural ITD information in amusic individuals. We measured psychophysical thresholds in lateralizing fixed-or varying-frequency musical tone pairs in amusics and matched control participants. In the second part of the study, we examined working memory for musical pitch sequence emanating from different ITD-simulated spatial locations. The aim is to determine whether working memory of the melodic sequence’s ITD-location feature is affected by concurrent variations along the pitch dimension of the sound sequence. If deficient pitch encoding impairs spatial ITD lateralization based on the hypothesized common underlying mechanism, we predict 1) higher thresholds in lateralizing the ITD in musical tone pairs in amusic individuals when pitches were varied and, 2) less recall of the sound sequence’s ITD information when concurrent pitch changes when involved than in fixed-pitch sequence in amusics. We report on an unimpaired temporal-fine-structure processing ability in individuals with congenital amusia in using ITD cues to lateralize musical pitch at the periphery. However, their ability to memorize the location and musical pitch of a sound sequence appears to be impaired and dissociated at the working memory level.

## Materials and methods

### Participants

Ten amusic individuals (six females, mean age = 26.3 years, *SD* = 2.2) and ten non-musically trained matched control participants (six females, mean age = 24.8 years, *SD* = 1.3) participated in this research. All participants had normal hearing and reported no history of psychiatric or neurological disorders. All participants were right-handed speakers of Mandarin Chinese. The two groups were comparable in age, level of education, and musical background or training (see Table 1). We used the Montreal Battery for the Evaluation of Amusia (MBEA; [10]) to screen participants for amusia. Participants who scored 2*SD* below the mean of the general population mean were classified as amusic [10, 37–40]. Table 2 showed the mean score and standard deviation on the scale, contour, interval, rhythm, metric, and memory subtests of the MBEA for the amusic and control groups as well as the global score. For individual MBEA subtest score, see supporting information (S1 File). A cutoff score of 23.1 on the global score (mean of the six subtests) was used as the criterion for diagnosis of congenital amusia. We selected to use the traditional accuracy cutoff rather than *d*-prime cutoff due to the more liberal criterion suggested for identification of amusic participants [37]. Participants signed written informed-consent forms and were paid for their participation. Note that the same amusic participants took part in all the experiments reported here. The experiment protocol was approved and conducted according to the guidelines of the Research Ethics Committee of National Taiwan University-Taiwan. Note that not all the research institutes here in Taiwan have an IRB affiliated with it, including National Central University (NCU). Therefore, all the research conducted here at NCU have to be submitted to the Research Ethics Committee at National Taiwan University (or other research institutes that have an affiliated IRB) for approval.

**Table 1:** Amusic and control group characteristics.

Characteristics	Amusics (N = 10)	Controls (N = 10)	p-value of t-test
**Demographic characteristics**			
*Age in years*	26.3 ± 2.2	24.8 ± 1.3	n.s.
*Gender*	6 female	6 female	*N/A*
*Musical education*	0.5 ± 0.4	0.7 ± 0.3	n.s.
**MBEA**			
*Total score*	21.7 ± 2.0	27.9 ± 1.2	*p* < 0.05

Values displayed represents mean ± 1 standard deviation.

**Table 2:** Mean score for each individual subtest of the MBEA and the global score for the amusic and control group.

	Scale	Contour	Interval	Rhythm	Metric	Memory	Global
**Amusic**							
*Mean (SD)*	21.5 (4.1)	23.0 (1.7)	20.5 (2.5)	23.0 (2.9)	17.8 (1.5)	24.4 (3.2)	21.7 (2.0)
**Control**							
*Mean (SD)*	26.9 (1.7)	27.5 (1.8)	26.8 (1.7)	27.8 (2.1)	26.0 (4.4)	28.4 (1.1)	27.9 (1.2)

The total score on each subtest is 30 points. Values displayed represents mean ± 1 standard deviation. The cutoff score of 23.1 on the global score **(**mean of the 6 subtests) was used as the criterion for diagnosis of congenital amusia.

### Task 1: ITD discrimination thresholds for tone pairs

#### Stimuli

Stimuli were generated using MATLAB software (MathWorks, Inc., Version 2009b) on an ASUS computer, and presented at a sampling rate of 44.1 kHz through 16-bit digital-to-analog converters (Creative Sound Blaster X-Fi Titanium). The experiments took place in a double-walled steel acoustically-isolated chamber (interior dimensions 2.0 m (L) × 2.0 m (W) × 2.5 m (H); Industrial Acoustics Company). All stimuli were presented through Sennheiser headphones (HD 380 Pro) at 70 dB SPL.

A pair of pure tones with frequency fixed at 261 Hz served as stimuli for the fixed-frequency tone pair condition (equivalent to C4 in Western music scale). There were five stimulus durations for a single tone in the fixed-frequency tone pair condition: 20, 50, 150, 250, and 500 ms. Each fixed-frequency tone pair was separated by a 500 ms within-pair silent interstimulus interval (ISI). For the varying-frequency tone pairs condition, pitch interval size was set at 0, 1, 5, and 10 semitones, which corresponds to tone-pair frequencies at 261/261, 261/277, 261/349, and 261/466 Hz, respectively. Two of the pitch interval sizes were larger than 2 semitones (i.e., above amusic individuals’ typical pitch discrimination threshold; [14, 33]). The duration of stimulus was fixed at 150 ms for all the varying-frequency pairs of tones based on results from fixed-frequency part of the study. All stimuli were ramped with a 10-ms linear rise-decay. A low-pass filtered noise with cutoff frequency at 1200 Hz was included to allow comparison with previous results covering ITD detection in amusics [26].

The spatial locations of the sounds were established by setting the ITDs between the left and right stereo channels of each tone. To generate the dichotic waveforms, the ITDs were set to zero in one randomly-chosen channel and to the desired interaural delay in the other channel using the following Eq (1). A positive ITD in this equation represents a waveform leading in time.

$$\begin{array}{l} X_{1}(t)=\sin\left(2\pi ft\right)\\ X_{2}(t)=\sin\left(2\pi f(t+ITD)\right) \end{array}$$ (1)

where *X*_*1*_ and *X*_*2*_ represents the two channels of the dichotic pure tone, *t* represents stimulus time, *f* represents stimulus frequency, and *ITD* represents interaural delay in microseconds.

#### Procedure

Difference limens were measured for lateralizing the ITDs in fixed-frequency and varying-frequency pairs of musical-tones using a 2-interval forced-choice (2IFC), 2-down 1-up adaptive design which tracked the participant’s 70.7% correct-response threshold [41, 42]. For the fixed-frequency tone-pair blocks, the order of tone durations (20, 50, 150, 250, and 500 ms) was randomly presented. Each participant completed 4 runs of 50 trials each per stimulus duration condition in a random-block design. For the varying-frequency tone-pair blocks, intervals of four different sizes (0, 1, 5, 10 semitones) were randomized across blocks. Each participant completed 5 runs of 50 trials each per stimulus condition in a random-block design.

**Fig 1:**
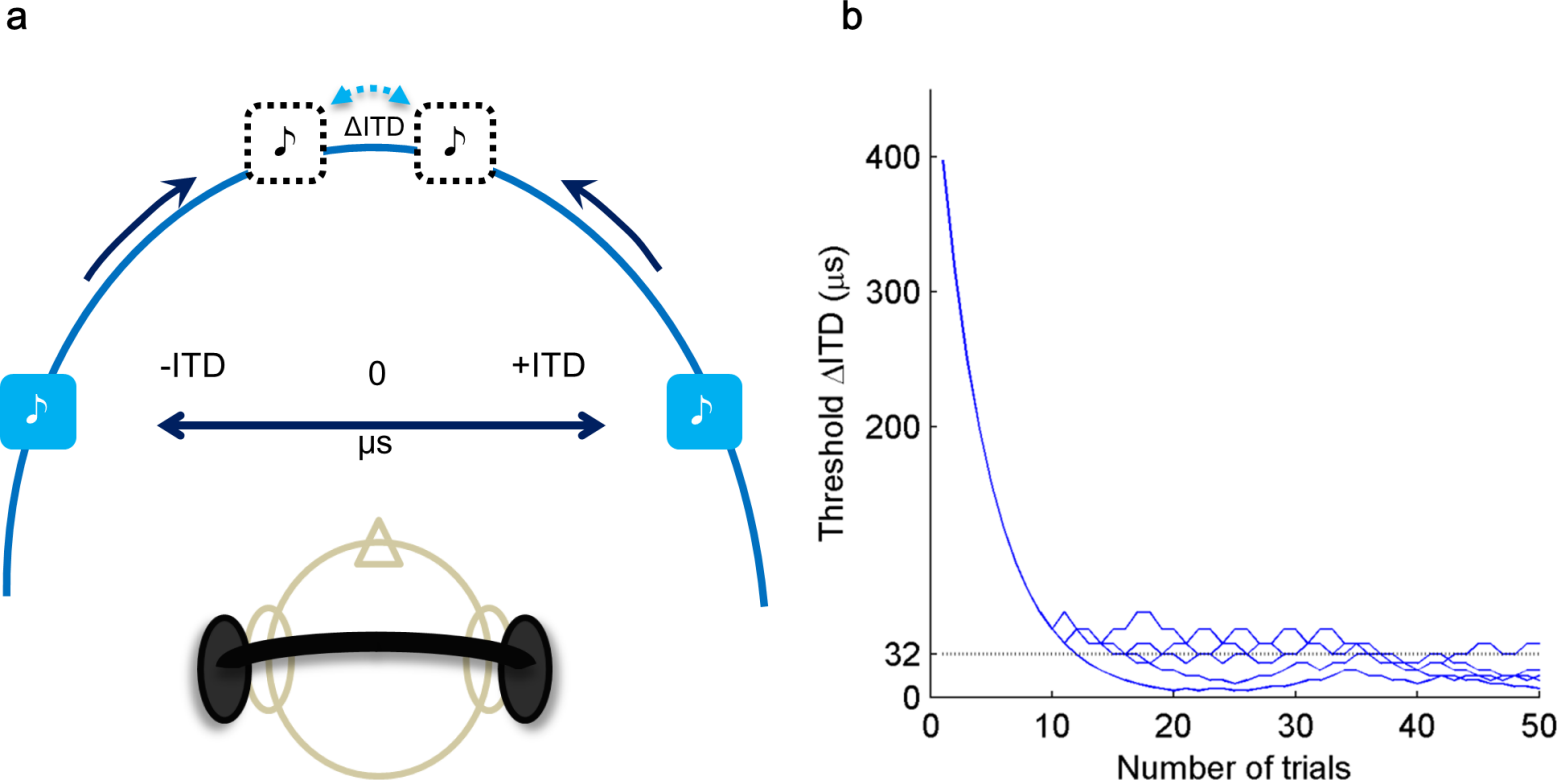
Schematic representation of Task 1 ITD lateralization procedure. (a) depicts lateralization task and (b) shows an example of ITD threshold tracking pattern from 1 participant. Dashed line (b) indicates ITD threshold averaged from 4 adaptive tracks.

On the first interval of each trial, the dichotic pure tone led to one randomly selected ear by a specific ITD, and in the second interval, it led to the other ear by the same magnitude of ITD. The participant’s task was to determine the location order of presentation of the tones (i.e., left leading, then right, or right leading, then left). Perceptually, this is equivalent to determining whether the two sounds in the two intervals of the trial were heard on the left, then the right, or the right, and then on the left. The participants then pressed either a left or a right key to respond (a left key response meant that they perceived the sound location as right to left). Participants received visual feedback after each trial, in the form of a plot of the staircase response (ITD as a function of trial number) shown on the monitor with a trial-by-trial update (Fig 1).

The initial value of the total ITD on each run was 400 μs (i.e., 200 μs in each interval). Two successive correct responses led to a reduction of the ITD by a step size of 0.1 log units [43]. An incorrect response led to an increase in ITD by the same step size. The threshold on each run was estimated as the average of the stimulus values at the reversal points. The first three or four reversals from each run were discarded, and the threshold was estimated as the average of the remaining even number of reversals. On average, six reversals went into the calculation of each threshold.

### Task 2: Memory for pitch and location within tone sequences

#### Stimuli

Each musical-note sequence contained five 250 ms notes randomly sampled from C4, E4, and G4 on the western music scale (equivalent to 262, 330, and 392 Hz) each separated by a 650 ms inter-tone interval (ITI). A 10 ms linear rise and decay ramp was applied to each stimulus. Each note within the sequence was created dichotically using the same equation as in Task 1 to simulate a randomly sampled ITD value at -650, 0, or 650 *μ*s corresponding to left, central, and right positions relevant to the vertical midline of the participant’s head.

#### Procedure

**Fig 2:**
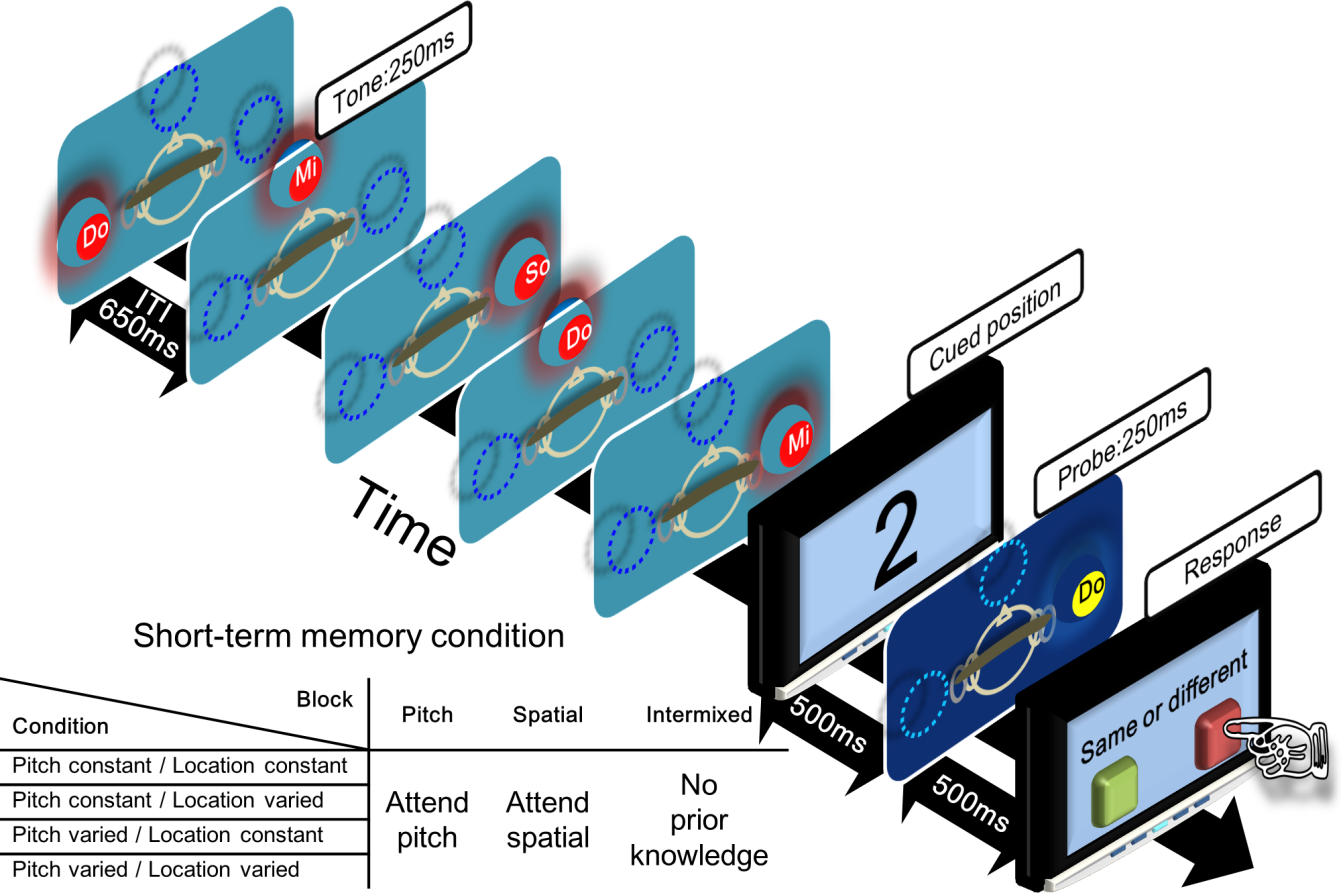
Schematic diagram of pitch and location probed recall working memory task. The sequence consisted of five 250ms musical tones with 650ms ITI. Cued position indicates the number-cued item in the sequence to be compared with the probe tone. Participants indicate whether the cued tone was the same or different with the probe with respect to the indicated feature (i.e., pitch, spatial).

For each trial, participants heard a five-tone sequence each originating from a different ITD-simulated locations presented via headphones. After 500 ms of silence, a number (from 1 to 5) probe appeared visually at the center of the screen to indicate the number-cued tone in the 5-note sequence to be compared. The probe sound was presented after 1000 ms interval following the termination of the last sound in the five-tone sequence. Participants compared the probe sound to the tone in the number-cued position in the sound sequence to see whether the relevant feature (pitch or location) matched with the probe sound. A schematic diagram of the experiment procedure is shown in Fig 2.

There were three different types of blocks (pitch, spatial, and intermixed) according to the to-be-encoded sound feature of the musical-note sequence. For spatial (i.e., ITD-location) blocks, participants were informed before each block of trials that they would be required to compare the spatial location (i.e., ITD-location, left, center, or right) to the probe tone with the number-cued position of the tone in the 5-tone sequence. Similarly, for the pitch blocks, participants were instructed before each block of trials to encode the pitch of the probed musical-note of the sound sequence. For the intermixed-blocks, participants were not informed before the block whether the feature to-be-compared would be the ITD or the pitch of the musical-note sequence. Instead, the task cue (pitch or spatial) appeared on the screen simultaneously with the number cue for the intermixed block condition. Participants had to compare the probe sound to number-cued sound in the preceding 5-tone sequence by the cued task condition (pitch or location). Participants pressed “D” key on the keyboard to indicate that the relevant task feature (spatial or pitch) of the probe was the same as the number-cued tone in the sequence, or pressed the “L” key to indicate that the relevant feature of the probe differed from the probe sound in the sequence.

**Fig 3:**
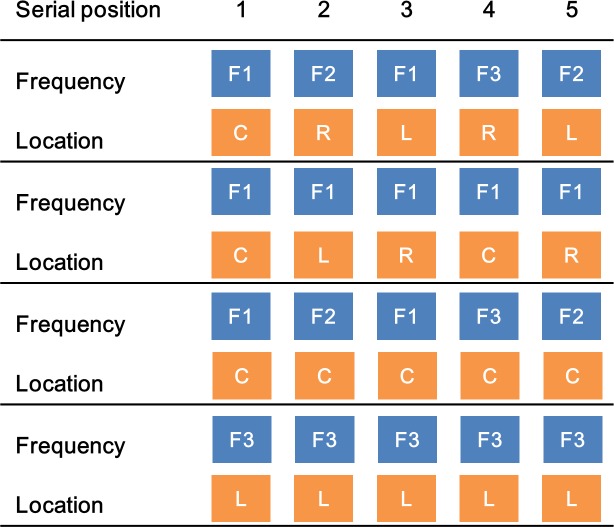
Schematic representation of the pitch/location stimulus sequence combination for working memory tasks. Frequency symbols F1, F2, F3 represent three different frequencies; location symbols C, R, L indicate sound lateralized to positions central, right, and left of participant’s head. Stimulus sequences from the top to bottom illustrate the four combinations of pitch and location constant or varied conditions.

Each block consisted of 160 trials total. In each block, there were four conditions of different difficulty levels with respect to encoding along either pitch or ITD dimensions, resulting in these condition combinations: constant pitch and constant location, constant pitch and varying location, varying pitch and constant location, and varying pitch and varying location. Each condition contained 40 trials × 4 combinations = 160 trials/block. Fig 3 displays an example of the four types of pitch/ITD combination conditions. Each participant completed a total of 4 blocks per each type of block (i.e., pitch, spatial, and intermixed). Block order was counterbalanced across participants. Before the actual experiment, each participant went through a 10-min practice session that contained 30 trials randomly sampled from all the different conditions. The total experiment took approximately 2 hrs to complete.

## Results

### Duration effects on ITD discrimination thresholds for tone pairs

**Fig 4:**
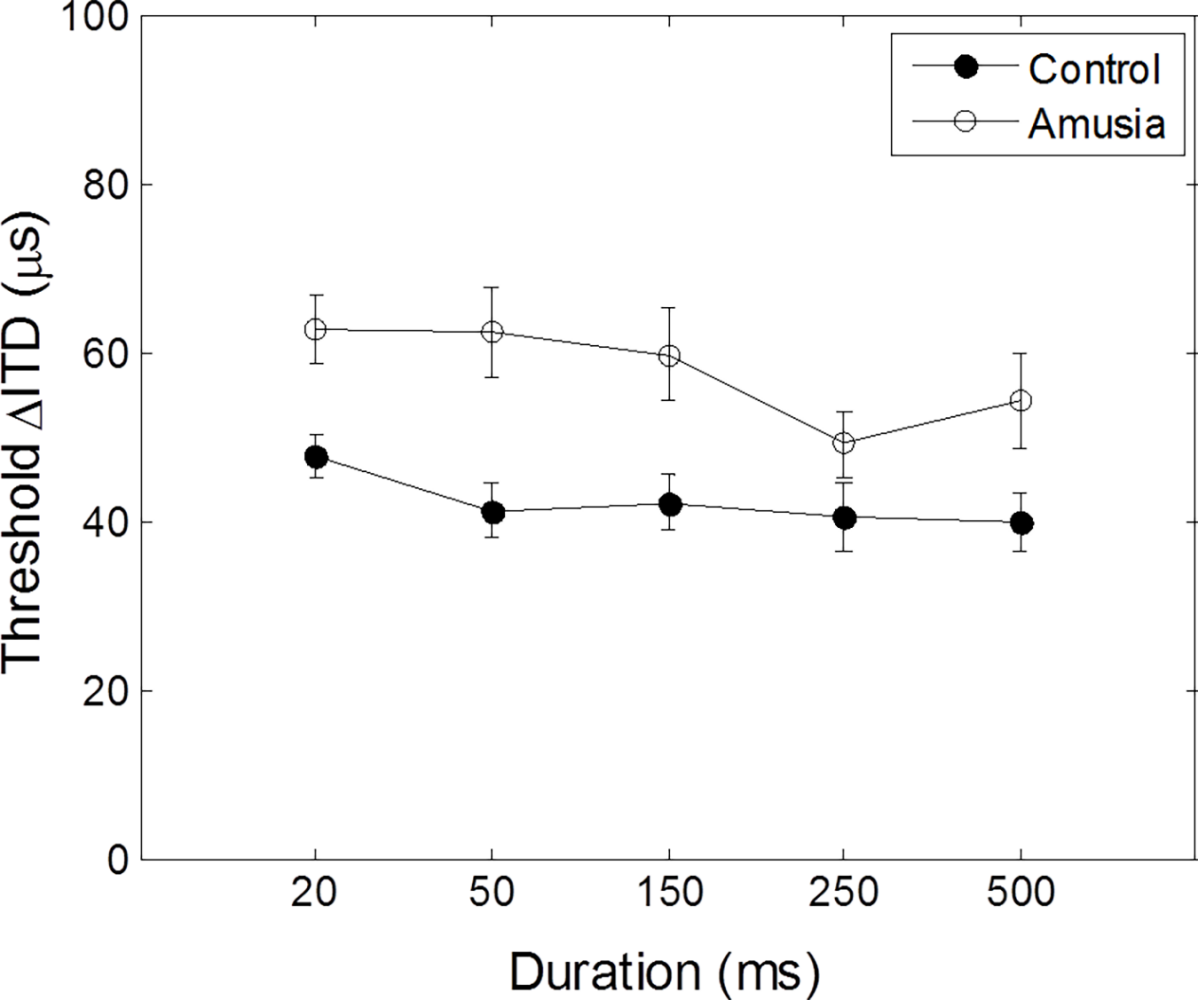
Mean ITD thresholds as a function of pitch durations for amusic and control participants. ITD thresholds for lateralizing fixed-frequency tone pairs were not significantly different for amusic and control groups. Error bars represent ± 1 standard deviation.

Fig 4 shows the mean ITD thresholds as a function of tone duration for amusics and control participants (S1 File). We carried out a 2 × 5 two-way mixed ANOVA with group (controls, amusics) as the between-subject factor and duration as the within-subject factor (20, 50, 150, 250, 500 ms). There was a significant main effect of tone duration, *F*(4, 72) = 2.71, *p* = 0.037. Linear trend analysis on tone duration was significant, *F*(1, 18) = 8.34, *p* = 0.01, indicating lateralization threshold decreased as tone duration increased. Post hoc pair-wise t-tests on tone duration revealed significance on 20 ms and 250 ms-tone only, *t*(19) = 2.672, *p* = 0.015. Importantly, however, ITD thresholds in lateralizing fixed-frequency musical pitch did not differ between amusic and matched-control groups, *F*(1, 18) = 1.90, *p* = 0.19. There was no significant interaction between tone duration and group, *F*(4, 72) = 0.864, *p* = 0.49.

### Pitch interval effects on ITD discrimination thresholds for tone pairs

**Fig 5:**
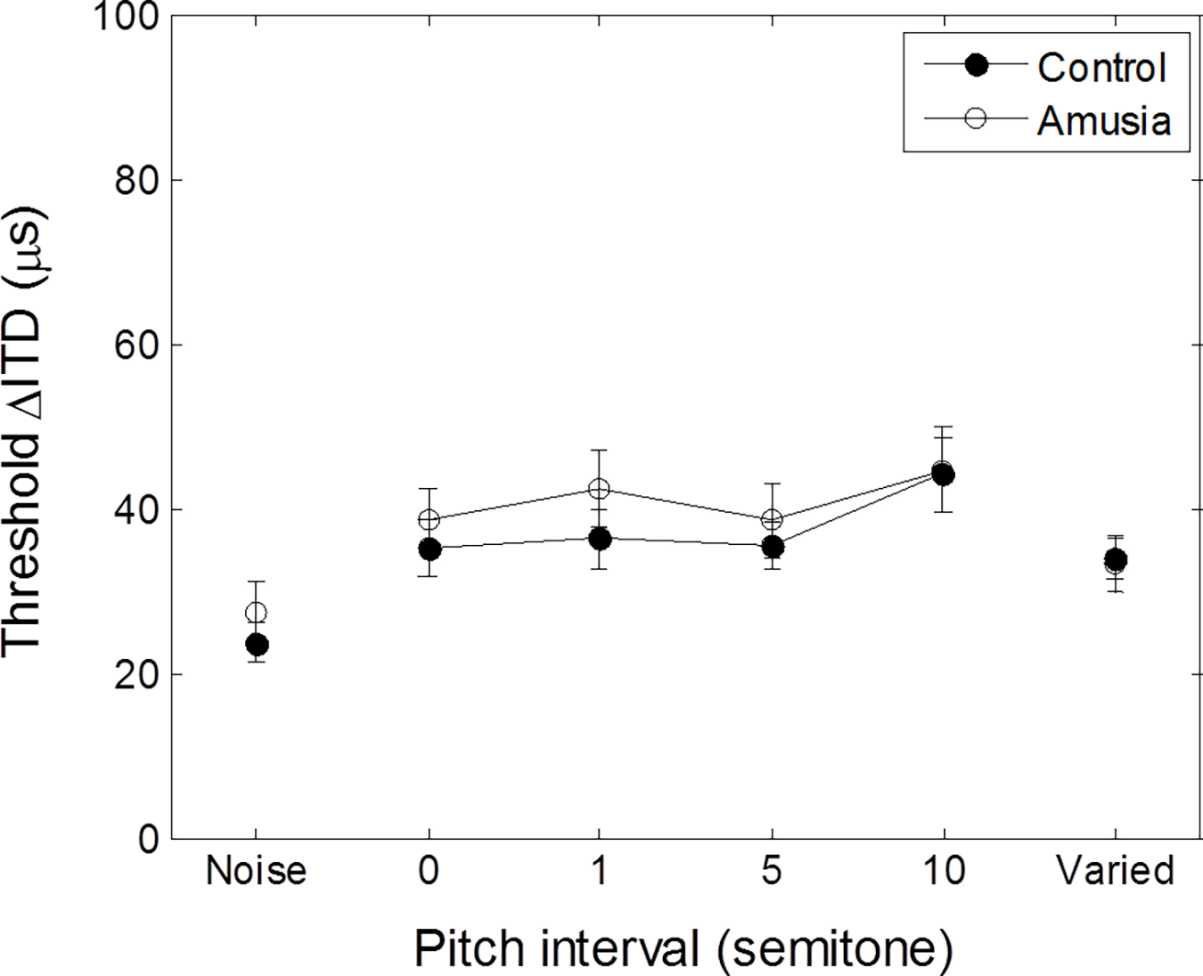
Mean ITD thresholds for lateralizing musical tone pairs with different pitch-interval size. The noise control condition used low-pass noise with cut-off frequency at 1200 Hz. In the varied condition, tone pairs were randomly selected from 0, 1, 5, 10 semitones. Size of pitch-interval had no effect on lateralizing musical tone pairs in control and amusic groups. Error bars represent ± 1 standard deviation.

Fig 5 shows the mean ITD thresholds for lateralizing musical tone pairs differing in pitch-interval size compared to low-pass noise condition for amusics and matched-controls (S1 File). We analyzed the threshold data in a two-way (2 × 6) mixed ANOVA with group (controls, amusics) as the between-subject factor and pitch interval as the within-subject factor (noise, 0, 1, 5, 10, varied). There was a significant main effect of pitch interval, *F*(3.41, 61.46) = 10.72, *p* < 0.001. Larger pitch intervals produced higher ITD thresholds. ITD thresholds in lateralizing musical tone-pairs separated by different semitones did not differ significantly between amusic and matched-control groups, *F* < 1.00. The interaction between pitch interval and group was not significant, *F* < 1.00. Post hoc comparison on ITD thresholds for lateralizing tone pairs with varied interval size compared to same-pitch pair (i.e., zero-interval) produced no significant difference, *t*(9) = 1.204, *p* = 0.26. ITD detection thresholds obtained in the low-pass noise condition compared to tone-pair conditions averaged across all tone-interval sizes revealed a significant difference, *t*(1) = −7.71, *p* < 0.001.

### Memory for pitch and location within tone sequences (informed condition)

**Fig 6:**
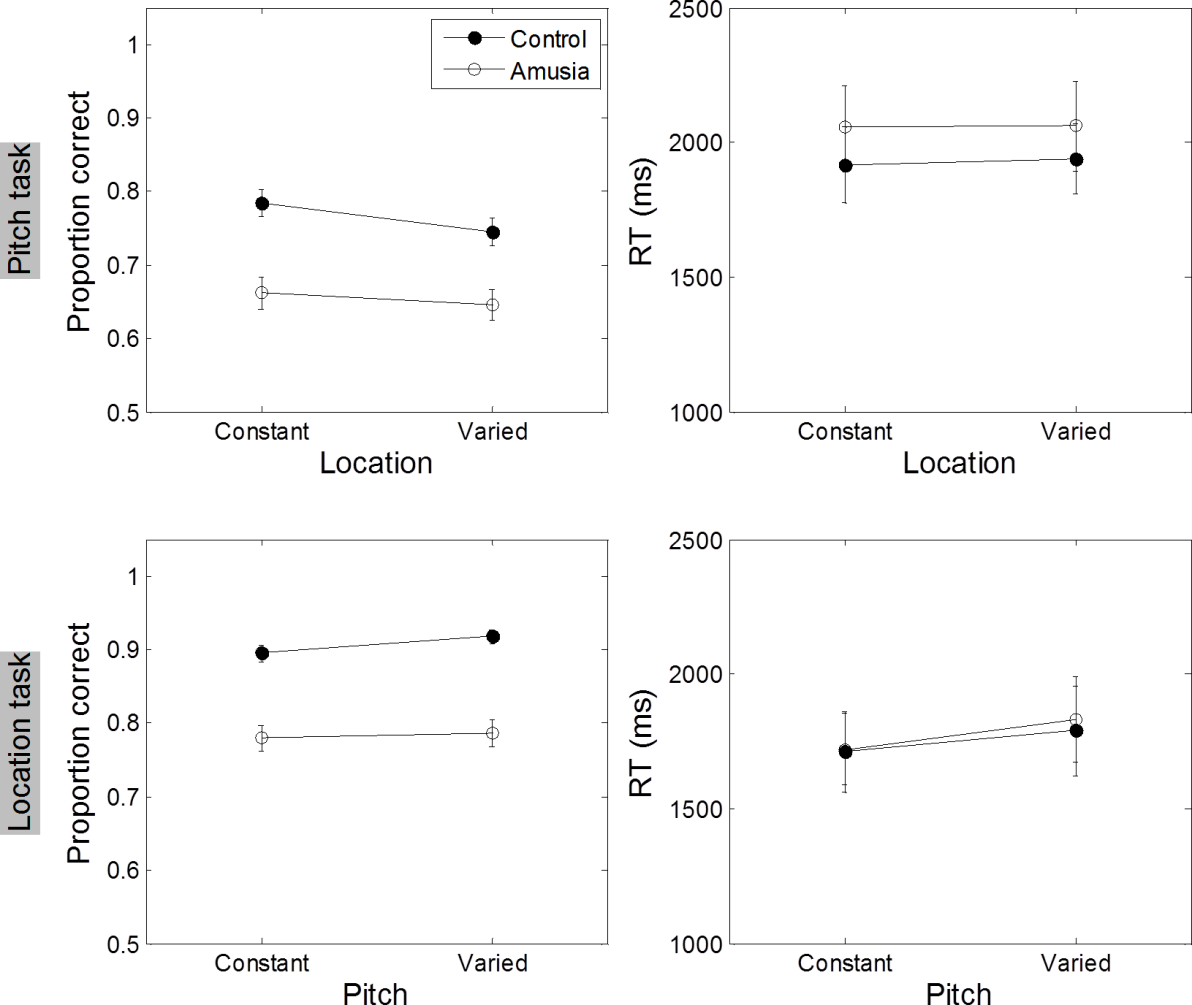
Mean accuracy and response time (RT) for working memory tasks (informed condition). Top panels: Mean proportion correct responses (left panel) and response times (right panel) when the probed recall feature was pitch as a function of location feature remains constant or varied. Bottom panels: Mean proportion correct responses (left panel) and response times (right panel) for the location task. Error bars represent ± 1 standard deviation.

To understand how working memory for the ITDs of musical-note sequence is affected by concurrent pitch feature of the tone sequence, the accuracy and response time in encoding ITDs when the pitch of the tone sequence remains constant or varied were analyzed. Fig 6 shows accuracy and response time (RT) for encoding pitch and ITD-location tasks for amusic and control groups (S2 File). The main effect for task revealed that recall accuracy for the location task was better than the pitch task across both groups, *F* (1, 18) = 11.205, *p* < 0.001. The amusic group exhibited impaired performance on working memory recall compared to controls irrespective of encoding the tone sequence’s pitch or location feature, *F*(1, 18) = 15.61, *p* = 0.001. There was no significant interaction between task and group. None of the planned comparisons testing the variation effect along either ITD or musical pitch dimension on accuracy in recalling the other dimension of the tone sequence reached significance, t(9) < 0.674, *p* > 0.34. The response time in recalling pitch or ITD did not differ when the to be encoded task information was pitch or location, *F*(1, 18) = 1.384, *p* = 0.25. We did not observe any difference between amusic and matched-control groups in the time it takes to recall pitch and ITD-location of tone sequences, *F*(1, 18) = 0.308, *p* = 0.59. The interaction between task and group was also nonsignificant, *F*(1, 18) = 0.885, *p* = 0.35.

### Memory for pitch and location within tone sequences (uninformed condition)

**Fig 7:**
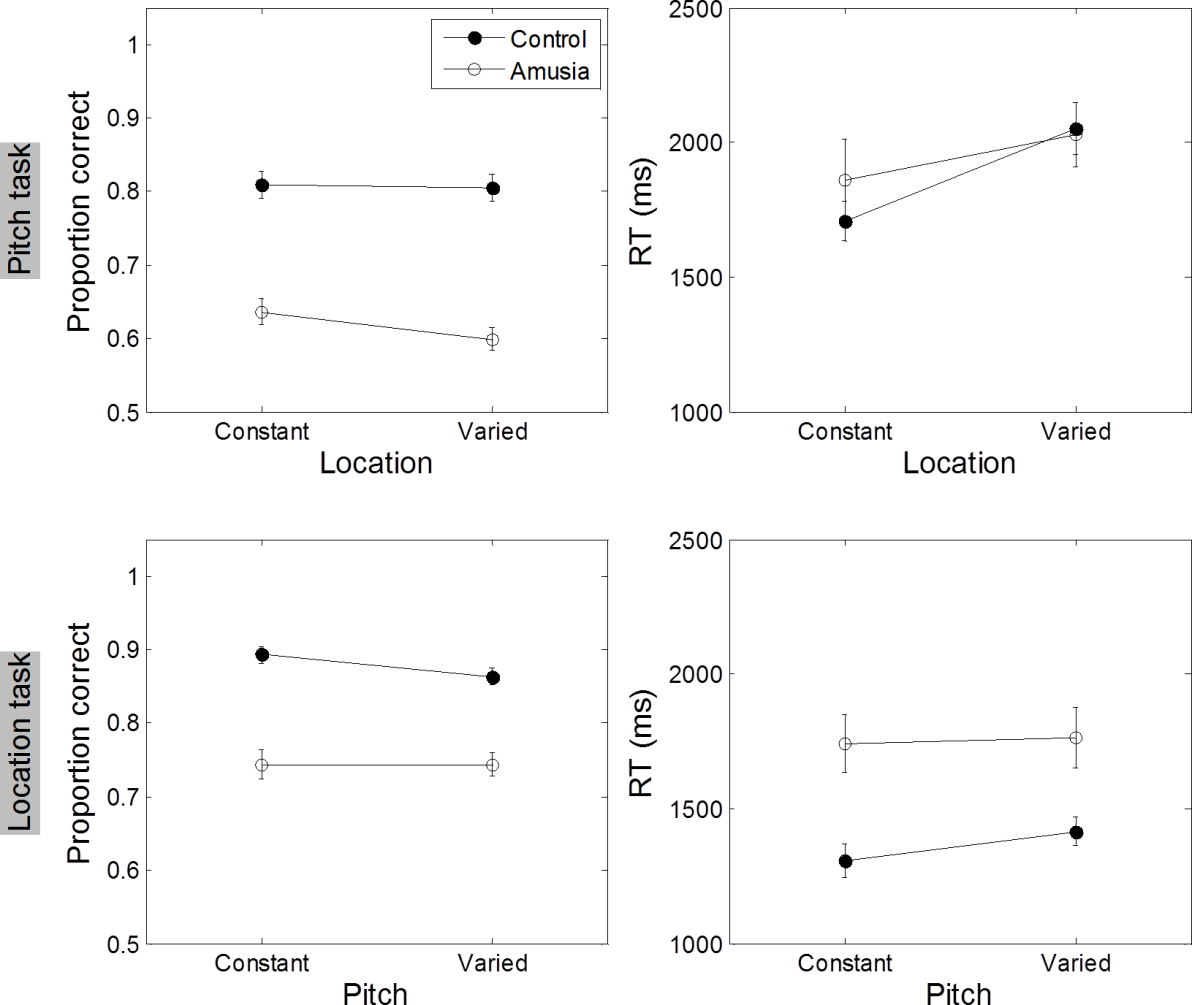
Mean accuracy and response time for working memory task (uninformed condition). Top panels: Mean proportion correct responses (left panel) and response times (right panel) when the probed recall feature was pitch as a function of location feature remains constant or varied. Bottom panels: Mean proportion correct responses (left panel) and response times (right panel) for the location task. Error bars represent ± 1 standard deviation.

To see whether recall accuracy would be affected when participants were uninformed of the to be remembered feature of the tone sequence, Fig 7 shows the accuracy and response time for encoding the pitch and ITD-location feature of tone sequence for amusic and control groups (S2 File). This plot revealed that control group had higher mean recall accuracy than the amusic group in encoding both pitch and ITD dimension of the tone sequence, *F*(1, 18) = 21.28, *p* < 0.001. Accuracy in recalling a probed ITD was significantly higher than recalling a probed pitch within the tone sequence for both groups, *F*(1, 18) = 13.62, *p* = 0.001. None of the planned comparisons showed significant effect of the other dimension information of the sequence on recall accuracy either for the pitch task, *t*(9) < 0.01, *p* > 0.99, nor for the location task, *t*(9) = 1.94, *p* = 0.08.

Analyses of response time (Fig 7 right) showed that it took participants longer time in recalling pitch than ITD feature of the sound sequence, *F*(1, 18) = 8.84, *p* = 0.008 Amusic participants took significantly more time to recall features of sounds than control participants, *F*(1, 18) = 8.43, *p* < 0.01. Planned comparisons showed that for control participants, RT mean differences between encoding location-varied and location-constant sequences in the pitch task reached significance, *t*(9) = −3.25, *p* = 0.009, but RTs for recalling ITD, whether pitch dimension was varied or not, did not differ significantly, *t*(9) = −1.30, *p* = 0.22. For the amusic group, variations along the other sound dimension did not significantly affect response time either in recalling pitch, *t*(9) = −0.96, *p* = 0.26, or recalling ITD information, *t*(9) = −0.46, *p* = 0.65.

## Discussion

Our results shed new light on how amusic individuals use the spectro-temporal fine structure of sounds to process the spatial location (ITDs) of musical tones at discrimination and working memory level. For discrimination tasks, amusic individuals showed similar ITD lateralization thresholds as typical listeners, even when lateralizing the position of varying musical-pitch intervals based on ITD cues. For working memory tasks, amusic individuals exhibited impaired performance relative to the control group in recalling both the pitch and spatial ITD-location of the musical tone sequences. Interestingly, variations along the pitch dimension of sound sequence did not affect memory performance in terms of accuracy in encoding the ITD-location of tone sequence for amusic and control groups. This may suggest an independence in processing the pitch and ITD features of sound sequence at working memory level.

Our findings demonstrate intact ITD discrimination ability in congenital amusics even when lateralizing the ITDs contained in varying musical pitch intervals. Amusic individuals showed compatible ITD detection thresholds as normal listeners [44–46] and exhibited improvements in ITD lateralization performance when tone duration was longer, consistent with ITD detection pattern reported for normal listeners [47, 48]. We did not observe pitch variations to have any effect on lateralizing the ITD-dependent location of musical tones, at least at the auditory peripheral level. Even when the pitch interval size were increased to more than 5 semitones (i.e., above amusic’s pitch discrimination threshold), there was no impairment in ITD detection threshold for the amusic group. It is valid to question why the putative effect of pitch interval size on lateralization not observed for matched controls should be observable in amusic individuals. The reasoning is as follows. Several previous studies have shown that amusic individuals exhibited worse performance than controls when a pitch distance larger than four semitones was employed in pitch change retention tasks [31–34]. One potential explanation was that when the stimuli with the same pitch interval (e.g., 4 semitones) were used for both groups, the stimuli would sound perceptually more similar for amusic individuals compared to controls, resulting in more difficult discrimination for the amusic group [34]. Therefore, even under identical pitch interval condition (e.g., 5 semitones), the perceptual (not physical) difficulty induced by pitch distance could account for the differences found between amusics and controls on pitch memory performance. Although in the current study we failed to observe a similar putative effect of pitch interval size on ITD lateralization, one reason could be due to the difference in the effect of perceptual pitch distance on memory and lateralization tasks. Another reason for this lack of effect could be that ITD processing is less susceptible to an influence of noisy pitch system as pitch interference effect have been reported in rhythm discrimination and temporal judgments among amusic individuals [21, 49]. Interestingly, studies have shown that amusic individuals exhibited normal metre discrimination when the beat stimuli did not involve variations in musical pitch [50]. This is consistent with the present findings of normal ITD detection thresholds for lateralizing fixed-frequency tone pairs and low-pass noise as the noise condition can be viewed as an “unpitched” situation. Similarly findings have also been reported in the emotional perception domain where amusics were able to recognize musical emotions based on temporal or consonance information of the musical excerpts [24].

The present findings on unimpaired use of temporal-fine structure of sound to code musical pitch’s ITD extends previous finding on fine ITD processing in noise for amusic individuals by showing that the ability to process the ITDs contained in musical tones were unaffected in amusic individuals. In addition, our results of fine auditory peripheral processing of spectro-temporal information are consistent with previous reports on normal pitch tracking mechanism up to the level of auditory cortex in amusics, but weak cortical neural representation of pitch to support reliable discrimination and memory [51]. In fact, abnormal neural transmission between the auditory cortex and right inferior frontal cortex of amusic individuals have been reported in several studies [51–56].

Findings on working memory of the pitch sequence’s ITD dimension showed that amusics’ impaired pitch perception did not affect memory for the location of sound objects based on ITD information. Manipulating the complexity along the ITD-location dimension did not influence accuracy in recalling the musical pitch of the sound object for amusic as well as for normal listeners. In other words, memory for the ITD-dependent locations of tone sequence was not affected by simultaneously encoding the tone’s pitch dimension, irrespective of whether one was informed of the to-be-recalled feature. Similar results have been reported in the visual domain, in which increasing the complexity of the visual-spatial pattern (on screen) had no effect on recall of the letters [57]. Our results extends previous findings by showing that in the auditory domain, ITD-dependent location and pitch seem to store separately at the working memory level even though both ITD and pitch rely on temporal phase locking mechanism at initial stage of auditory processing [5]. Such dissociation of working memory storage and retrieval for pitch and binaural ITDs is in line with the notion that musical pitch is stored separately from other perceptual features [58].

Regarding overall feature processing, our results showed that sound object’s ITD location was more easily recalled than pitch feature for both amusic and control groups. One possibility for the difference in recall accuracy could be due to different rehearsal mechanisms for pitch and location features. Whereas location information might be readily rehearsed verbally as right, left or center positions, the musical pitch dimension (i.e., pure tones) does not lend itself easily to assignment of verbal labels as in consonants or pictures (except for people with absolute pitch). An alternative explanation could be the specificity of reference point used to rehearse location and musical pitch features for recall. Several studies have shown that most people are quite accurate when required to sing the first few notes of a familiar melody, suggesting that almost all humans have some degree of “absolute” pitch memory to classify pitch as high or low [59, 60]. Thus one may conceive that musical pitch feature may be rehearsed verbally as higher or lower just as location information can be rehearsed as left and right. The difference in recall accuracy between the localization and pitch task can thus be contributed to the specificity of reference point. While most people probably use the body midline as a consistent reference point in the spatial localization task to classify left and right, the reference point for musical pitch may be more susceptible to individual variability or between-trial adjustments. In addition, the use of a relative label for pitch or spatial location becomes more difficult if the number of items to be memorize increases or the proximity between pitch or location items decreases. For both ITD and pitch memory tasks, normal listeners showed better memory performance than amusic individuals. This finding is in line with several psychophysical reports showing an impaired and easily distracted memory for musical pitch in individuals with amusia [33–35]. Interestingly, amusic individuals exhibited impaired memory for the sound object’s ITD simulated spatial-location memory even though the amusics’ ITD discrimination thresholds at the perceptual level was compatible to that of normal listeners.

The results of working memory tasks suggest that musical pitch and spatial location of the auditory objects are retained and processed in two separate streams in auditory working memory. Impaired pitch system observed in individuals affected with congenital amusia did not interfere with memory of the spatial locations of musical pitch sequence. Manipulating the complexity level of the musical pitch dimension had no effect on recall performance of the spatial location of the pitch sequence, supporting a dissociation of processing between pitch and ITD features of auditory objects at working memory level. Our finding is consistent with recent EEG and MEG evidence showing a topographical difference between sound frequency and spatial location processing in auditory working memory [61]. In addition, the independent processing of pitch and ITD features observed here is consistent with the putative model of auditory dorsal and ventral streams for processing spatial and non-spatial properties of sounds, respectively [62, 63]. Since we observed a lack of interference between pitch and ITD features of sound here, one could infer that perhaps no binding or no robust binding exist between these two sound features. However, this did not completely rule out the possibility that a weak binding exists between pitch and ITD features of sound in working memory among amusic individuals. Future studies that manipulate the difficulty levels of the pitch or ITD dimension of tone sequences, or tailor the perceptual difficulty of pitch interval stimuli based on an individual’s pitch discrimination threshold could further shed light on the issue of feature binding in auditory working memory.

## Conclusions

The present study showed that individuals affected by congenital amusia are unimpaired in extracting the temporal fine-structure of sounds to lateralize the location of musical pitches using ITD information in the auditory periphery and at working memory level. Concurrent variations along the pitch feature of the sound object did not interfere with peripheral ITD discrimination. At the working memory level, amusic individuals showed impaired recall of both the musical pitch and ITD-dependent location features of the sound object. Increasing the complexity level along either sound feature had no effect on processing of the other feature, suggesting independent processing of pitch and ITD features of sound in working memory. Consistent with the dorsal and ventral account of segregated processing streams, the present findings showed no evidence of feature binding between musical pitch and ITD-dependent location in auditory working memory. Our findings demonstrate fine peripheral signal encoding and provide further evidence of a dissociative, but impaired, pitch and ITD encoding process at the auditory working memory level in amusic individuals.

## Supporting information

S1 File MBEA score and Expt. 1 data. Used for Figs 4 & 5.
(XLSX)

S2 File Expt. 2 data. Used for Figs 6 & 7.
(XLSX)

## References

[1] Bernstein LR. Auditory processing of interaural timing information: new insights. J Neurosci Res. 2001;66(6):1035–46. 10.1002/jnr.10103 11746435

[2] Grothe B, Park TJ. Sensitivity to interaural time differences in the medial superior olive of a small mammal, the Mexican free-tailed bat. J Neurosci. 1998;18(16):6608–22. 9698347 10.1523/JNEUROSCI.18-16-06608.1998 PMC6793185

[3] Joris PX, Yin TCT. Responses to amplitude‐modulated tones in the auditory nerve of the cat. J Acoust Soc Am. 1992;91(1):215–32. 1737873 10.1121/1.402757

[4] Weiss TF, Rose C. A comparison of synchronization filters in different auditory receptor organs. Hearing Res. 1988;33(2):175–9. 10.1016/0378-5955(88)90030-5 3397327

[5] Furukawa S, Washizawa S, Ochi A, Kashino M. How independent are the pitch and interaural-time-difference mechanisms that rely on temporal fine structure information? In: Moore BCJ, Patterson RD, Winter IM, Carlyon RP, Gockel HE, editors. Basic aspects of hearing: Physiology and perception. New York, NY: Springer New York; 2013 pp. 91–9. 10.1007/978-1-4614-1590-9_11 23716213

[6] Meddis R, Hewitt MJ. Virtual pitch and phase sensitivity of a computer model of the auditory periphery. I: Pitch identification. J Acoust Soc Am. 1991;89(6):2866–82.

[7] Meddis R, Hewitt MJ. Virtual pitch and phase sensitivity of a computer model of the auditory periphery. II: Phase sensitivity. J Acoust Soc Am. 1991;89(6):2883–94.

[8] Oxenham AJ. Pitch perception. J Neurosci. 2012;32(39):13335–8. 10.1523/JNEUROSCI.3815-12.2012 23015422 PMC3481156

[9] Peretz I. The biological foundations of music: Insights from congenital amusia In: Deutsch D, editor. The psychology of music. 3rd ed San Diego, CA: Elsevier; 2013 pp. 551–64.

[10] Peretz I, Champod AS, Hyde K. Varieties of musical disorders: the Montreal Battery of Evaluation of Amusi. Ann NY Acad Sci. 2003;999(1):58–75. 14681118 10.1196/annals.1284.006

[11] Peretz I, Vuvan DT. Prevalence of congenital amusia. European Journal Of Human Genetics. 2017;25:625 10.1038/ejhg.2017.15 28224991 PMC5437896

[12] Drayna D, Manichaikul A, de Lange M, Snieder H, Spector T. Genetic correlates of musical pitch recognition in humans. Science. 2001;291(5510):1969–72. 10.1126/science.291.5510.1969 11239158

[13] Peretz I, Cummings S, Dubé M-P. The genetics of congenital amusia (tone deafness): a family-aggregation study. J Hum Genet. 2007;81(3):582–8. 10.1086/521337 PMC1950825 17701903

[14] Hyde KL, Peretz I. Brains that are out of tune but in time. Psychol Sci. 2004;15(5):356–60. 10.1111/j.0956-7976.2004.00683.x 15102148

[15] Liu B. Uncertain risk analysis and uncertain reliability analysis. J Uncertain Sys. 2010;4(3):163–70.

[16] Whiteford KL, Oxenham AJ. Learning for pitch and melody discrimination in congenital amusia. Cortex. 2018;103:164–78. 10.1016/j.cortex.2018.03.012 29655041 PMC5988957

[17] Tillmann B, Albouy P, Caclin A. Congenital amusias. Handb Clin Neurol. 2015;129:589–605. 10.1016/B978-0-444-62630-1.00033-0 25726292

[18] Foxton JM, Dean JL, Gee R, Peretz I, Griffiths TD. Characterization of deficits in pitch perception underlying ‘tone deafness’. Brain. 2004;127(4):801–10. 14985262 10.1093/brain/awh105

[19] Ayotte J, Peretz I, Hyde K. Congenital amusia: a group study of adults afflicted with a music‐specific disorder. Brain. 2002;125(2):238–51. 11844725 10.1093/brain/awf028

[20] Dalla Bella S, Giguère J-F, Peretz I. Singing in congenital amusia. J Acoust Soc Am. 2009;126(1):414–24. 10.1121/1.3132504 19603898

[21] Foxton JM, Nandy RK, Griffiths TD. Rhythm deficits in ‘tone deafness’. Brain Cogn. 2006;62(1):24–9. 10.1016/j.bandc.2006.03.005 16684584

[22] Peretz I, Ayotte J, Zatorre RJ, Mehler J, Ahad P, Penhune VB, et al Congenital amusia: a disorder of fine-grained pitch discrimination. Neuron. 2002;33(2):185–91. 11804567 10.1016/s0896-6273(01)00580-3

[23] Tillmann B, Lévêque Y, Fornoni L, Albouy P, Caclin A. Impaired short-term memory for pitch in congenital amusia. Brain Res. 2016;1640:251–63. 10.1016/j.brainres.2015.10.035 26505915

[24] Gosselin N, Paquette S, Peretz I. Sensitivity to musical emotions in congenital amusia. Cortex. 2015;71:171–82. 10.1016/j.cortex.2015.06.022 26226563

[25] Jiang C, Liu F, Wong PC. Sensitivity to musical emotion is influenced by tonal structure in congenital amusia. Sci Rep. 2017;7(1):7624 10.1038/s41598-017-08005-x 28790442 PMC5548738

[26] Cousineau M, Oxenham AJ, Peretz I. Congenital amusia: a cognitive disorder limited to resolved harmonics and with no peripheral basis. Neuropsychologia. 2015;66:293–301. 10.1016/j.neuropsychologia.2014.11.031 25433224 PMC4300951

[27] Vuvan DT, Nunes-Silva M, Peretz I. Meta-analytic evidence for the non-modularity of pitch processing in congenital amusia. Cortex. 2015;69:186–200. 10.1016/j.cortex.2015.05.002 26079675

[28] Douglas KM, Bilkey DK. Amusia is associated with deficits in spatial processing. Nat Neurosci. 2007;10(7):915–21. 10.1038/nn1925 17589505

[29] Tillmann B, Jolicœur P, Ishihara M, Gosselin N, Bertrand O, Rossetti Y, et al The amusic brain: lost in music, but not in space. PLoS One. 2010;5(4):e10173 10.1371/journal.pone.0010173 20422050 PMC2858073

[30] Williamson VJ, Cocchini G, Stewart L. The relationship between pitch and space in congenital amusia. Brain Cogn. 2011;76(1):70–6. 10.1016/j.bandc.2011.02.016 21440971

[31] Albouy P, Cousineau M, Caclin A, Tillmann B, Peretz I. Impaired encoding of rapid pitch information underlies perception and memory deficits in congenital amusia. Sci Rep. 2016;6:18861 10.1038/srep18861 26732511 PMC4702148

[32] Gosselin N, Jolicœur P, Peretz I. Impaired memory for pitch in congenital amusia. Ann NY Acad Sci. 2009;1169(1):270–2. 19673791 10.1111/j.1749-6632.2009.04762.x

[33] Williamson VJ, Stewart L. Memory for pitch in congenital amusia: beyond a fine-grained pitch discrimination problem. Memory. 2010;18(6):657–69. 10.1080/09658211.2010.501339 20706954

[34] Jiang C, Lim VK, Wang H, Hamm JP. Difficulties with pitch discrimination influences pitch memory performance: evidence from congenital amusia. PLoS One. 2013;8(10):e79216 10.1371/journal.pone.0079216 24205375 PMC3808300

[35] Williamson VJ, McDonald C, Deutsch D, Griffiths TD, Stewart L. Faster decline of pitch memory over time in congenital amusia. Adv Cogn Psychol. 2010;6:15–22. 10.2478/v10053-008-0073-5 20689638 PMC2916665

[36] Albouy P, Mattout J, Bouet R, Maby E, Sanchez G, Aguera P-E, et al Impaired pitch perception and memory in congenital amusia: the deficit starts in the auditory cortex. Brain. 2013;136(5):1639–61. 23616587 10.1093/brain/awt082

[37] Vuvan DT, Paquette S, Mignault Goulet G, Royal I, Felezeu M, Peretz I. The Montreal Protocol for Identification of Amusia. Behavior Research Methods. 2018;50(2):662–72. 10.3758/s13428-017-0892-8 28455794

[38] Henry MJ, McAuley JD. Failure to Apply Signal Detection Theory to the Montreal Battery of Evaluation of Amusia May Misdiagnose Amusia. Music Percept. 2013;30(5):480–96.

[39] Henry MJ, McAuley JD. On the Prevalence of Congenital Amusia. Music Percept. 2010;27(5):413–8.

[40] Pfeifer J, Hamann S. Revising the diagnosis of congenital amusia with the Montreal Battery of Evaluation of Amusia. Frontiers in Human Neuroscience. 2015;9(161). 10.3389/fnhum.2015.00161 25883562 PMC4381621

[41] Wetherill GB, Levitt H. Sequential estimation of points on a psychometric function. Brit J Math Stat Psy. 1965;18(1):1–10. 10.1111/j.2044-8317.1965.tb00689.x 14324842

[42] Levitt H. Transformed up‐down methods in psychoacoustics. J Acoust Soc Am. 1971;49(2B):467–77. 5541744

[43] Saberi K. Some considerations on the use of adaptive methods for estimating interaural‐delay thresholds. J Acoust Soc Am. 1995;98(3):1803–6. 7560514 10.1121/1.413379

[44] Brown AD, Kuznetsova MS, Spain WJ, Stecker GC. Frequency-specific, location-nonspecific adaptation of interaural time difference sensitivity. Hearing Res. 2012;291(1):52–6. 10.1016/j.heares.2012.06.002 PMC3416959 22732693

[45] Hartmann WM, Dunai L, Qu T. Interaural time difference thresholds as a function of frequency In: Moore BCJ, Patterson RD, Winter IM, Carlyon RP, Gockel HE, editors. Basic aspects of hearing: Physiology and perception. New York, NY: Springer New York; 2013 pp. 239–46. 10.1007/978-1-4614-1590-9_27 23716229

[46] Wright BA, Fitzgerald MB. Different patterns of human discrimination learning for two interaural cues to sound-source location. Proc Natl Acad Sci USA. 2001;98(21):12307–12. 10.1073/pnas.211220498 11593048 PMC59810

[47] Moore BCJ. An introduction to the psychology of hearing Bingley, UK Emerald; 2012.

[48] Tobias JV, Zerlin S. Lateralization threshold as a function of stimulus duration. J Acoust Soc Am. 1959;31(12):1591–4.

[49] Pfeuty M, Peretz I. Abnormal pitch—time interference in congenital amusia: evidence from an implicit test. Atten Percept Psycho. 2010;72(3):763–74. 10.3758/APP.72.3.763 20348581

[50] Phillips-Silver J, Toiviainen P, Gosselin N, Peretz I. Amusic does not mean unmusical: Beat perception and synchronization ability despite pitch deafness. Cognitive Neuropsychology. 2013;30(5):311–31. 10.1080/02643294.2013.863183 24344816

[51] Peretz I. Neurobiology of Congenital Amusia. Trends in Cognitive Sciences. 2016;20(11):857–67. 10.1016/j.tics.2016.09.002 27692992

[52] Hyde KL, Zatorre RJ, Griffiths TD, Lerch JP, Peretz I. Morphometry of the amusic brain: a two-site study. Brain. 2006;129(10):2562–70. 16931534 10.1093/brain/awl204

[53] Hyde KL, Lerch JP, Zatorre RJ, Griffiths TD, Evans AC, Peretz I. Cortical thickness in congenital amusia: when less is better than more. J Neurosci. 2007;27(47):13028–32. 10.1523/JNEUROSCI.3039-07.2007 18032676 PMC6673307

[54] Mandell J, Schulze K, Schlaug G. Congenital amusia: an auditory-motor feedback disorder? Restor Neurol Neurosci. 2007;25(3–4):323–34. 17943009

[55] Loui P, Alsop D, Schlaug G. Tone-deafness–a new disconnection syndrome? J Neurosci. 2009;29(33):10215–20. 10.1523/JNEUROSCI.1701-09.2009 19692596 PMC2747525

[56] Hyde KL, Zatorre RJ, Peretz I. Functional MRI evidence of an abnormal neural network for pitch processing in congenital amusia. Cereb Cortex. 2011;21(2):292–9. 10.1093/cercor/bhq094 20494966

[57] Guérard K, Morey CC, Lagacé S, Tremblay S. Asymmetric binding in serial memory for verbal and spatial information. Mem Cognit. 2013;41(3):378–91. 10.3758/s13421-012-0275-4 23254536

[58] Berz WL. Working Memory in Music: A Theoretical Model. Music Percept. 1995;12(3):353.

[59] Frieler K, Fischinger T, Schlemmer K, Lothwesen K, Jakubowski K, Müllensiefen D. Absolute memory for pitch: A comparative replication of Levitin’s 1994 study in six European labs. Musicae Scientiae. 2013;17(3):334–49.

[60] Levitin DJ. Absolute memory for musical pitch: Evidence from the production of learned melodies. Perception & Psychophysics. 1994;56(4):414–23. 7984397 10.3758/bf03206733

[61] Kaiser J. Dynamics of auditory working memory. Frontiers in Psychology. 2015;6:613 10.3389/fpsyg.2015.00613 26029146 PMC4426685

[62] Alain C, Arnott SR, Hevenor S, Graham S, Grady CL. “What” and “where” in the human auditory system. Proceedings of the National Academy of Sciences of the United States of America. 2001;98(21):12301–6. 10.1073/pnas.211209098 11572938 PMC59809

[63] Kaiser J, Lutzenberger W. Induced Gamma-Band Activity and Human Brain Function. The Neuroscientist. 2003;9(6):475–84. 10.1177/1073858403259137 14678580

